# Prevalence and risk factors of mental disorders among refugees and internally displaced persons in the MENA Region: a systematic literature review

**DOI:** 10.1192/j.eurpsy.2026.11631

**Published:** 2026-07-31

**Authors:** F. Zaouali, I. Anes

**Affiliations:** 1 Emergency department and mobile emergency and resuscitation service, Tahar Sfar University Hospital, Mahdia; 2 Outpatient psychiatric consultation, Hadj Ali Soua Regional Hospital, Ksar Hellal, Monastir, Tunisia

## Abstract

**Introduction:**

The Middle East and North Africa (MENA) region has been deeply affected by conflicts and humanitarian crises, leading to millions of refugees and internally displaced persons (IDPs). These populations are exposed to multiple stressors that predispose them to high rates of mental health disorders.

**Objectives:**

This review aims to systematically assess the prevalence and risk factors of mental health disorders among refugees and IDPs in the MENA region, synthesizing current evidence to inform healthcare strategies.

**Methods:**

A systematic literature search was conducted in PubMed, Scopus, and PsycINFO databases covering the period from January 2000 to April 2025. Search terms included combinations of keywords related to “refugees,” “internally displaced persons,” “mental health,” “PTSD,” “depression,” “anxiety,” and “MENA countries” (including specific country names such as Palestine, Syria, Iraq, Lebanon, Jordan, etc.). Inclusion criteria were: (1) original research articles; (2) cross-sectional or cohort studies; (3) reporting prevalence rates of mental health disorders among refugees or IDPs; (4) conducted within MENA countries or involving populations from this region; (5) published in English or French. Exclusion criteria included qualitative-only studies, reviews, case reports, or studies outside the geographic focus. Two independent reviewers screened titles, abstracts, and full texts. Data extracted included sample size, population characteristics, diagnostic tools, prevalence rates, and associated risk factors. The quality of included studies was assessed using the Newcastle-Ottawa Scale adapted for cross-sectional studies.

**Results:**

Out of 512 records initially identified, 32 studies met the inclusion criteria, encompassing a total of 21,659 participants. The included studies were conducted mainly in Syria, Lebanon, Jordan, Iraq, and Turkey. Commonly used diagnostic tools included the Harvard Trauma Questionnaire (HTQ), the Patient Health Questionnaire (PHQ-9), and the Generalized Anxiety Disorder Scale (GAD-7). The pooled prevalence of depression was 42% (95% CI: 30%–54%), anxiety 43% (95% CI: 31%–57%), PTSD 45% (95% CI: 36%–53%), and stress disorders 22% (95% CI: 11%–39%). Female gender, unemployment, and unmarried status were significant risk factors. Several studies highlighted barriers to mental health care including stigma, limited access, and lack of culturally adapted services.

**Conclusions:**

Mental health disorders are highly prevalent among refugees and IDPs in the MENA region, necessitating urgent and culturally sensitive mental health services integrated within broader humanitarian responses. Future studies should emphasize intervention effectiveness and strategies to mitigate stigma and enhance mental health literacy.

**Disclosure of Interest:**

None Declared

